# The role of Chito-oligosaccharide in regulating ovarian germ stem cells function and restoring ovarian function in chemotherapy mice

**DOI:** 10.1186/s12958-021-00699-z

**Published:** 2021-01-25

**Authors:** Yaoqi Huang, Haifeng Ye, Feiyin Zhu, Chuan Hu, Yuehui Zheng

**Affiliations:** 1 Department of Obstetrics & Gynecology, the Second Affiliated Hospital of Nanchang University, Nanchang, Jiangxi China; 2 Comprehensive Pneumology Center, Institute of Lung Biology and Disease, Helmholtz Zentrum München, Munich, Germany; 3 Jiangxi Medical College, Nanchang University, Nanchang, Jiangxi China; 4 Department of reproductive health, Shenzhen Traditional Chinese Medicine Hospital, Shenzhen, Guangdong China

**Keywords:** Chemotherapy, OGSCs, COS, Inflammation

## Abstract

In recent years, the discovery of ovarian germ stem cells (OGSCs) has provided a new research direction for the treatment of female infertility. The ovarian microenvironment affects the proliferation and differentiation of OGSCs, and immune cells and related cytokines are important components of the microenvironment. However, whether improving the ovarian microenvironment can regulate the proliferation of OGSCs and remodel ovarian function has not been reported. In this study, we chelated chito-oligosaccharide (COS) with fluorescein isothiocyanate (FITC) to track the distribution of COS in the body. COS was given to mice through the best route of administration, and the changes in ovarian and immune function were detected using assays of organ index, follicle counting, serum estrogen (E_2_) and anti-Mullerian hormone (AMH) levels, and the expression of IL-2 and TNF-α in the ovaries. We found that COS significantly increased the organ index of the ovary and immune organs, reduced the rate of follicular atresia, increased the levels of E_2_ and AMH hormones, and increased the protein expression of IL-2 and TNF-α in the ovary. Then, COS and OGSCs were co-cultured to observe the combination of COS and OGSCs, and measure the survival rate of OGSCs. With increasing time, the fluorescence intensity of cells gradually increased, and the cytokines IL-2 and TNF-α significantly promoted the proliferation of OGSCs. In conclusion, COS could significantly improve the ovarian and immune function of chemotherapy model mice, and improve the survival rate of OGSCs, which provided a preliminary blueprint for further exploring the mechanism of COS in protecting ovarian function.

## Introduction

Ovarian dysfunction includes a “natural” decline in ovarian function caused by age factors and pathological ovarian function decline caused by pathogenic factors such as radiotherapy, chemotherapy and surgery. Menopause is regarded as a landmark event. Its essence is ovarian follicle depletion, and the resulting low estrogen levels in the ovary induce the occurrence and development of many other diseases [1]. Women under 40 years of age, who have hypomenorrhea or amenorrhea for at least 4 months, and whose follicle stimulating hormone (FSH) level are greater than 25 U/L, are defined as premature ovarian failure(POF )[2, 3]. Because of the unclear pathogenesis, estrogen and progestogen replacement therapy are currently used to improve other diseases caused by low estrogen levels, however, adverse reactions are more frequent, and the effects are not good.

In 2004, Johnson [4] and others found that the atresia rate of mouse follicles was significantly higher than the decrease of non-atretic follicles in follicle counting. It was found that ovoid cells in the epithelial layer of the ovary simultaneously expressed the germ cell specific marker MVH and the proliferation of cell marker BrdU using double-immunofluorescence staining. This discovery invalidated the “fixation theory of primordial follicle pool” and suggested for the first time the hypothesis that germ stem cells also exist in the ovary. Niikura [5] and others observed germ cells expressing Stra8 (stimulated by retinoic acid gene 8) in the ovarian surface epithelium, indicating that there may be germ cells with meiotic function in the ovary. Subsequently, Wu et al. [6] successfully isolated OGSCs from the ovaries of pups and adult mice for the first time, transfected them with GFP virus in an appropriate environment in vitro, and then transplanted them into the ovaries of immunodeficient mice. Normal GFP-positive offspring were produced after mating with male mice. At present, it is currently suspected that OGSCs may come from the ovarian cortex, which expresses stem cell markers and have the ability to develop into oocytes [7–9]. Some scholars have proposed that the loss of OGSCs in vivo and abnormal function may be related to ovarian aging [10–13]. It is worth noting that the proliferation and differentiation of stem cells cannot be separated from the surrounding microenvironment, and immune system related cells are an important part of the microenvironment of the OGSCs nest [14]. When OGSC nests are destroyed, ovarian dysfunction occurs [15, 16]. Some studies have shown that infertility may occur in neonatal mice with thymectomy [17]. In short, once the mechanism of OGSCs has been thoroughly studied, it might bring new hope for the clinical treatment of infertility patients caused by chemotherapy.

COS is the only basic amino oligosaccharide in natural sugar. It is derived mainly from small molecular oligosaccharides with amino groups that are degraded after deacetylation of chitosan from crustaceans such as shrimp and crab. COS is formed by polymerization of 2–10 identical or different monosaccharides by glycosidic bonds, and its content in nature is second only to cellulose [18–20]. It has been confirmed that COS administration by intraperitoneal injection and intragastric administration significantly improved the phagocytic function of mouse peritoneal macrophages, increased the index of immune organs such as thymus and spleen, stimulated lymphocytes to secrete IL-2 cytokines, and enhanced the activity of NK cells [21, 22]. COS stimulated the secretion of other cytokines by activating macrophages, to then produce a cascade reaction [23]. Several studies showed that it improves the body’s immunity and play the role of antioxidant, and is also recognized as an immune enhancer [24]. By observing the effect of COS on the reproductive capacity of male mice, some scholars found that COS significantly improved the reproductive capacity of male mice, improved the antioxidant capacity of testes, and promoted sperm formation [25]. However, it remains unclear whether the immunopotentiation of chitooligosaccharides can improve the ovarian microenvironment, promote the proliferation of OGSCs and reshape ovarian function.

In this study, we used a chemical modification method to connect fluorescein isothiocyanate (FITC) and COS to form stable FITC-COS labeled products, which were analyzed using high performance liquid chromatography [26, 27]. Then FITC-COS was given to mice in various ways, and the best way to reach the ovary was screened using a spectrophotometer [28–30]. We also constructed a mouse model of CY/BUS-induced gonadotoxicity, and then we administered COS to mice to evaluate the changes in ovarian function and immune function in mice, and to further study whether COS can directly regulate the immune factors IL-2 and TNF - α to promote the proliferation of OGSCs, to gain a new understanding of ovarian dysfunction with limited therapeutic effects.

## Materials and methods

### Animals and treatment

In this experiment, clean-grade Kunming female mice aged 4 weeks, weighing 16 –25 g, and female Kunming suckling mice aged 3–5 days were selected and purchased from the Medical Animal Center of Nanchang University. Animal certificate number: SYXK (Gan) 2010–0002. Mice aged 4 weeks were randomly divided into the oral gavage group, intraperitoneal injection group and tail vein injection group. According to the sampling time, each group was divided into eight time periods: 0.25 h, 0.5 h, 1 h, 2 h, 4 h, 8 h, 12 h and 24 h. Eight mice were randomly assigned to each time period, and were fasted for 12 h before the experiment. Then, FITC-COS solution was administered to mice by different administration methods (10 mg/mL, 0.5 mL). When the mice were sacrificed at the corresponding times, the ovaries were immediately removed and washed with normal saline; the surface was dried with filter paper, weighed, and made into tissue homogenates according to the corresponding proportion. The supernatants were centrifuged at 6000 rpm for 10 min to measure the absorbance. In addition, Kunming female mice were randomly divided into the blank control group, saline group, and chitooligosaccharide treatment group (COS group) after modeling. A model of infertility was established by intraperitoneal injection of cyclophosphamide (120 mg/kg)/busulfan (30 mg/kg) according to the references. The control group and saline group were given the same amount of saline continuously, and the COS treatment group was given 300 mg/kg.d COS continuously by the best administration method. The body weight of mice was recorded weekly, and the indexes of ovary, thymus and spleen (the ratio of the weight of bilateral ovaries, spleen and thymus to their body weight respectively) was calculated after 28 days of treatment. Under sterile conditions, suckling mice aged 3–5 days were sacrificed, ovaries were extracted, ovarian appendages were carefully removed, and intact ovaries were washed in D-Hanks solution for later use. The temperature of the mouse house was controlled at 22 ± 2 °C, the relative humidity was maintained at approximately 50%, ensuring 12 h light and 12 h darkness, and the mice had free access to food and drinking water. All experiments were carried out according to the guidelines of the Institutional Animal Ethics Committee (IAEC) of Nanchang University (Nanchang, P. R. China).

### OGSC primary isolation and culture

Ten to sixteen ovaries of 3-to 5-day-old postnatal mice were collected and placed in D-Hanks solution to remove surrounding fat tissue. The OGSCs were isolated using the modified two-step enzymatic method [31, 32]. The intact ovarian tissue was removed by forceps and placed into 15 mL falcon tubes with collagenase IV digestion solution (1 mg/mL, Sigma, USA). Under the condition of avoiding light, the falcon tube is placed in a 37 °C water bath, gently shaken in one direction, and the digestion time is about 13 min. The solution was centrifuged for 10 min and removes the supernatant, and 0.05% trypsin solution (Sigma, USA) containing EDTA (1 mM, Sigma, USA) was added to the falcon tube for resuspending, which was fully shaken and kept in a constant temperature water bath at 37 °C for 3 min. In addition, DMEM cell culture medium containing 10% fetal bovine serum was prepared to stop trypsin digestion, in subsequent experiments at 2000 rpm, centrifuged for 10 min and supernatants were removed. The isolated OGSCs were then cultured in minimum essential medium alpha (MEM-α; Invitrogen), 10% (v/v) FBS (Gibco), 1 mM sodium pyruvate (Gibco), 1 mM non-essential amino acids (Gibco), 2 mM L-glutamine (Sigma), 0.1 mM β-mercaptoethanol (Sigma), 20 ng/mL mouse leukemia inhibitory factor (LIF) (Sigma), 10 ng/mL mouse epidermal growth factor (EGF) (Sigma), 40 ng/mL mouse glial cell line-derived neurotrophic factor (GDNF) (Sigma), 1 ng/mL basic fibroblast growth factor (bFGF) (Sigma) and 100 × penicillin and streptomycin (P/S) (Solarbio). After standing for 3 s, when the ovary was about to descend to the bottom of the falcon tube, the cell suspension was added to the 48-well cell culture plate, and the remaining ovarian tissue was transferred into another well. After adding the culture medium again, the cell plate was put into a 37 °C 5% CO_2_ incubator for further culture.

### FITC standard curve

A certain amount of FITC powder was accurately weighed, and dissolved in anhydrous methanol, then 1 mg/ml FITC methanol solution was prepared, which was shaken and mixed evenly. Then 0.1 mol/l acetic acid was prepared to gradually dilute FITC methanol solution into six series of standard FITC solutions with concentrations of 0.001 mg/ml, 0.004 mg/ml, 0.006 mg/ml, 0.008 mg/ml, 0.01 mg/ml and 0.016 mg/ml, and then mixed well and kept away from light. The fluorescence intensity awas determined at an excitation wavelength of 485 nm and an emission wavelength of 535 nm. The absorbance value was used as the ordinate of the standard curve and the FITC concentration (mg/ml) was used as the abscissa.

### Labeling of chitooligosaccharides

50 mL of FITC- anhydrous methanol solution (1 mg/mL) and 20 mL of COS aqueous solution (15%, m/V) were prepared, and the pH value of the COS aqueous solution was adjusted to 9. This solution was thoroughly mixed and magnet-stirred 3–24 h at room temperature in the dark (to ensure that the mass ratio of COS to FITC was controlled at 60: 1). The solution was then poured into dialysis bags and dialyzed with ddH_2_O for 5 days until the solution outside the dialysis bag was found to be nonfluorescent. Then, the dialyzed solution was washed with excess ethanol three times (5–6 times the volume of anhydrous ethanol was slowly poured in). When no fluorescence absorption was detected in the supernatant, the liquid was freeze-dried in vacuum to obtain the labeled product FITC-COS, which was stored away from light. FITC-COS powder was prepared into a 1 mg/ml FITC-COS methanol solution, and then diluted to various multiples using 0.1 mol/L acetic acid solution, and the absorbance of the solution was determined. The absorbance value was substituted into the FITC-COS standard curve to calculate the content of FITC in the FITC-COS sample using the formula: Labeling rate(%) = FITC(mg)/FITC-COS (mg) × 100%, the labeling rate of the sample was calculated.

### Chromatographic analysis of FITC-COS

FITC standard solution and FITC-COS solution of 0.1 μg/mL were prepared for chromatographic analysis. Chromatographic model: Agilent 1260 Infinity;

Chromatographic column: Venusil AA (4.6 mm × 250 mm,5 μm); Mobile phase: Phase A was 0.095 mol/L sodium acetate acetonitrile solution (pH = 6.5), phase B was 80% acetonitrile, and gradient elution was performed(0–10 min, 100% A; 10–12 min, linear gradient 100% A → 70% A; 12–15 min, linear gradient 70% → 50%; 15–18 min constant gradient 0% A; 18–25 min, 100% A), the flow rate was 0.8 ml/min, and the column temperature was 30 °C. The detection wavelength was 490 nm and the injection volume was 10 μL.

### Precision, accuracy and stability of FITC-COS

FITC-COS was freeze-dried, and dissolved in a 0.1 mol/L acetic acid solution and prepared into an initial solution with a concentration of 1 mg/mL. Then, 0.1 mol/L acetic acid was further diluted to standard solutions of different concentrations. Normal mouse ovarian tissue (10 mg) and PBS 600 μL were added to the standard solution, and the tissue homogenate was fully ground and centrifuged at 6500 rpm for 10 min. The supernatants were taken to determine the fluorescence intensity A value, and the standard liquid concentration was taken as the X-axis and absorbance A as the Y-axis. The linear regression equation was calculated and the curve was drawn. The absorbance of FITC-COS solution (25 μg/mL) was measured immediately and after 1 week of storage (in the dark at 4 °C), the light absorption values of the two were compared. An ovarian tissue homogenate solution containing FITC-COS (25 μg/mL) was also prepared. The absorbance was measured immediately and at room temperature for 4 h, and the absorbance was compared. FITC-COS tissue samples were prepared with low (0.78 μg/mL), medium (12.5 μg/mL) and high (50 μg/mL) concentrations. In the same operation as above, the absorbance A value was determined, and then the concentration of FITC-COS in each sample solution was obtained by plugging it into the associated standard curve, and the accuracy rate (%) was divided by the added concentration. Five samples were measured for each concentration, and the experiment was repeated three times to calculate the intrabatch precision and interbatch precision.

### Detection of serum estrogen and anti-Mullerian hormone

The estrous cycle of mice was observed by vaginal smear. After anesthesia, blood was taken from the eyeball and left for 30 min. After centrifugation at 3500 rpm, the supernatant was taken and the hormone levers were measured by ELISA kit.

### HE staining and follicle counting

The ovaries were soaked overnight in 4% paraformaldehyde fixative solution. Ovaries then were embedded in HistoGel and paraffin and serially sectioned (5 μM). All the sections were stained with hematoxylin and eosin using standard methods. Primordial follicles contained an oocyte in the center surrounded by a single layer of squamous granulosa cells, primary follicles contained an oocyte surrounded by a single layer of cuboidal granulosa cells, secondary follicles contained an oocyte surrounded by 6–12 layers of cuboidal granulosa cells and theca cell layers, antral follicles contained an oocyte surrounded by multiple layers of cuboidal granulosa cells with a fluid-filled antral space and theca cell layers. The numbers of follicles of each specific type were counted in every fifth serial section as previously described and the percentages of primordial and atretic follicles were calculated [33–35]. The morphological changes of ovaries were observed under 200X magnification.

### Reverse transcription- PCR

According to the manufacturer’s instructions, total RNA was extracted from OGSCs by the Trizol method(Invitrogen,Germany), and the concentration and purity of total RNA were detected by a nanometer photometer(IMPLEN, Germany). The measured optical density was 1.8 < A260/A280 < 2.2.RNA expression was detected by 2% agarose gel electrophoresis(Bio-Rad, USA). Table 1 contains the list of primers used in this study.

**Table 1:** Sequences used for Reverse Transcription- PCR

Gene	Forward primer (5′-3′)	Reverse primer (5′-3′)
GAPDH	AACGGATTTGGCCGTATTGG	CATTCTCGGCCTTGACTGTG
MVH	GTGTATTATTGTAGCACCAACTCG	CACCCTTGTACTATCTGTCGAACT
Fragilis	CTGGTCCCTGTTCAATACACTCTT	CAGTCACATCACCCACCATCTT
OCT-4	AGCTGCTGAAGCAGAAGAGG	GGTTCTCATTGTTGTCGGCT
Stella	CAGTCTACGGAACCGCATTG	CTTGGGAAAGGCGCTTTGAA
Dazl	GCCCTGCAATCAGGAAACAA	GGTTGGAGGCTGCATGTAAG
c-Kit	AACAGGACCTCGGCTAACAA	ACTGGCATCAGAGTTGGACA
ZP3	CTCTCCAGTTCACGGTGGAT	CGACTTTGAGATGGCAGGTG
Scp3	GCCGCTGAGCAAACATCTAA	GGCTTCCCAGATTTCCCAGA
Figla	AGCAGGAAGCCCAGTAAAGT	GCTCCTCAGGGCTTTGTTTC

### Immunoblotting and immunofluorescence

Western blotting and immunofluorescence were performed according to the operation manual. The primary antibodies used in this study included MVH (Abcam, ab27591), OCT4 (Abcam, ab18976), GAPDH (Abcam, ab181602), TNF-α (Abcam, ab6671), IL-2 (Abcam, ab11510). The EdU kit was purchased from Keygen BioTECH (KGA337–1000). All HRP and fluorophore-conjugated secondary antibodies were obtained from Affinity Biosciences. After incubation with the secondary antibody, imprints were imaged using an EasySee Western blot kit (DW101–01, TransGen Biotech). Images AI600 and ImageJ were used to scan and analyze the images.

### Alkaline phosphatase staining

Cells were removed from the CO_2_ incubator, washed carefully and slowly with PBS, and fixed with 4% paraformaldehyde for 15 min. We discarded 4% paraformaldehyde, washed with PBS twice, added 200 μL ALP staining reagent (Solarbio, G1480) under the condition of avoiding light, and incubated it at room temperature for 15 min. The liquid was discarded, and the petri dish was washed with PBS 3 times. Then, 200 μL PBS solution was added to the petri dish, and photos were taken under the microscope.

### Cell proliferation assay

The cell suspension was evenly tiled onto 96-well cell plates (100 μL/well) and cultured in an incubator at 37 °C and 5% CO_2_ for 24–72 h to make the cells adherent to the wall. Then, 100 μL of drugs of different concentrations (0 ng/mL, 1 ng/mL, 5 ng/mL, 10 ng/mL and 20 ng/mL) were added to each well cell, and each group was cultured for 72 h. Before adding CCK-8, we replaced fresh culture medium to remove the influence of the drug, added 10 μL of CCK-8 solution (Transgen BioTECH, China) to each well, and incubated in the incubator in the dark for 1 h. The absorbance OD value was measured at a wavelength of 450 nm.

### Laser confocal observation of the distribution

FITC-COS was dissolved in ultrapure water and filtered using a 0.2 μm filter membrane. Chitosan oligosaccharide (50 μg/mL) and ovarian germline stem cells were cultured in a CO_2_ incubator for 10 min, 30 min, 1 h and 2 h. The culture dish was removed from the CO_2_ incubator, washed twice with PBS, and new OGSC culture medium was added, which was stored away from light. The images were observed and collected under a laser confocal microscope as soon as possible.

### Statistical analysis

The experimental results were statistically analyzed using Graph Pad Prism 7.0. Statistical differences between sets of data were detected using one-way ANOVA. All data were expressed as the mean ± standard deviation of at least three independent experiments. *P* < 0.05 was regarded as statistically significant, and *P* < 0.01 was considered extremely statistically significant.

## Results

### Preparation and chromatographic analysis of FITC- chitosan oligosaccharides

**Fig. 1:**
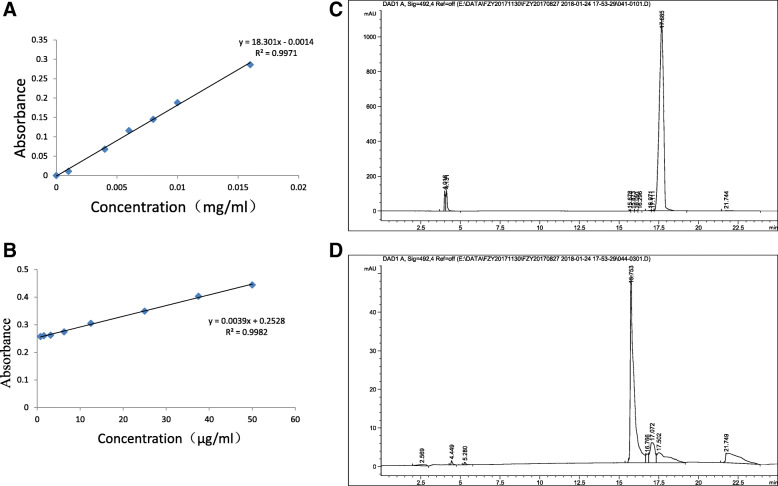
Preparation and chromatographic analysis of FITC- chitosan oligosaccharides **a** The standard curve of FITC solution; **b** Typical standard calibration curve for the determination of FITC-COS in the ovary. **c** HPLC chromatograms of FITC; **d** HPLC chromatograms of FITC-COS

We plotted the standard curve of the FITC solution. According to the experimental results, the standard curve of the FITC solution had a good linear relationship, in the range of 0.001–0.016 mg/ml, and the *R*-value was 0.9985. According to the curve, the labeling rate of FITC-COS measured was 3.5% (Fig. 1a). The FITC-COS standard curve of the ovarian tissue measured had a good linear relationship in the range of 0.78–50 μg/mL, and the *R*-value was 0.9991 (Fig. 1b). According to the HPLC chromatogram results, the prepared chitosan oligosaccharides and FITC were combined after a sufficient reaction, the retention time was 15.753 min (Fig. 1d), and the retention time of FITC was 17.685 min (Fig. 1c). Figure 1d has no peak in the period of 17.685 min, indicating that the FITC-COS preparation did not have free luciferin, which can exclude the interference effect of free FITC luciferin in subsequent experiments.

### Precision, accuracy and stability of FITC-COS in ovaries

The intrabatch precision, interbatch precision and accuracy of the ovarian tissue homogenates containing FITC-COS are shown in Table 2. Single-factor analysis of variance (ANOVA) was used to calculate intrabatch precision and interbatch precision. Among them, the coefficient of intrabatch variation was 1.90–4.05%, and the coefficient of interbatch variation was 2.23–5.33%. Both values were less than 10%. The accuracy of FITC-COS with high, medium and low concentrations in the ovarian samples was measured as 99.53–104.42%, and the results were all within the range of 80–120%. Therefore, this experiment conforms to the pharmacokinetic study of drug distribution detection in vivo. The stability of the FITC standard solution and the ovarian sample solution containing FITC-COS after being placed at room temperature for a period of time is shown in Table 3. The results showed that the fluorescence values of the newly configured solutions were changed, but the RSD was less than 10%.

**Table 2:** Precision and accuracy for analysis of FITC-COS in ovary(*n* = 3)

Sample	Concentration (μg/mL)	Intra-assay RSD (%)	Inter-assay RSD (%)	RE (%)
Ovary	0.78	4.05	5.33	104.42
	12.5	2.51	3.06	103.37
	50.00	1.90	2.23	99.53

**Table 3:** Stablities of standard solution and ovary sample solution(*n* = 3)

Sample	RSD (%)
The standard solution for FITC	0.47
Prepared ovary samples	4.47

**Table 4:** Effect of COS on viscera index in mice

Group	Ovary	Spleen	Thymus
	Index (‰)	Index (‰)	Index (‰)
Control	0.581 ± 0.012	3.501 ± 0.179	2.705 ± 0.019
CY/BUS	0.289 ± 0.015***	2.552 ± 0.054**	1.881 ± 0.036***
CY/BUS+COS	0.439 ± 0.016^##^	3.302 ± 0.115^##^	2.340 ± 0.038^###^

** *P* < 0.01, and ***P < 0.001, vs. the control group, ^##^*P* < 0.01 and ^###^*P* < 0.001 vs. the CY/BUS group

### The distribution of chitooligosaccharides in the ovary

**Fig. 2:**
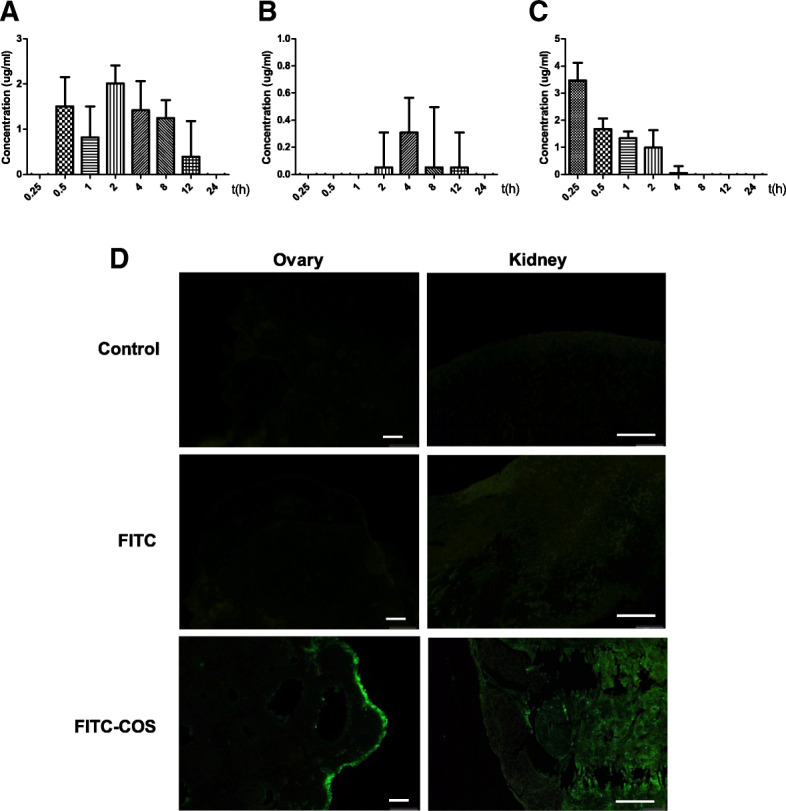
The distribution of chitooligosaccharides in the ovary. **a**-**c** The distribution of chitosan oligosaccharide in ovary at different time after intraperitoneal injection, gavage administration and tail vein injection; **d** Distribution of ovaries and kidneys after intraperitoneal injection. Scale bar = 100 μm

Mice in each group were sacrificed at each time period, and the ovarian tissue was removed to measure the drug concentration. The measured results are shown in Fig. 2. The retention time of intraperitoneal injection was the longest in the ovaries, and fluorescence was detected from 0.5 to 12 h (Fig. 2a). The administration of chitosan oligosaccharide through tail vein injection takes the fastest time to reach the ovaries; however, the retention time of the ovaries is not as long as that of intraperitoneal injection, and the administration method was more difficult (Fig. 2c). Only a small portion of the chitosan oligosaccharides reached the ovaries by gavage administration and the retention time was relatively short (Fig. 2b). In summary, the intraperitoneal injection was the best method for the delivery of chitooligosaccharides to the ovaries. To further verify the distribution of chitosan oligosaccharides in ovaries after intraperitoneal administration, the fluorescence labeled chitosan oligosaccharides were distributed in the ovarian cortex through tissue embedding and paraffin sections, while in the kidney group, chitooligosaccharides were distributed in the parenchyma, and no fluorescence was observed in the unlabeled chitooligosaccharide control group (Fig. 2d). In order to explore whether FITC adheres to cells in a non-specific way, we injected FITC and normal saline into mice as a control. The results showed that FITC did not adhere to cells.

### Protective effects of chitooligosaccharides on reproductive and immune function

**Fig. 3:**
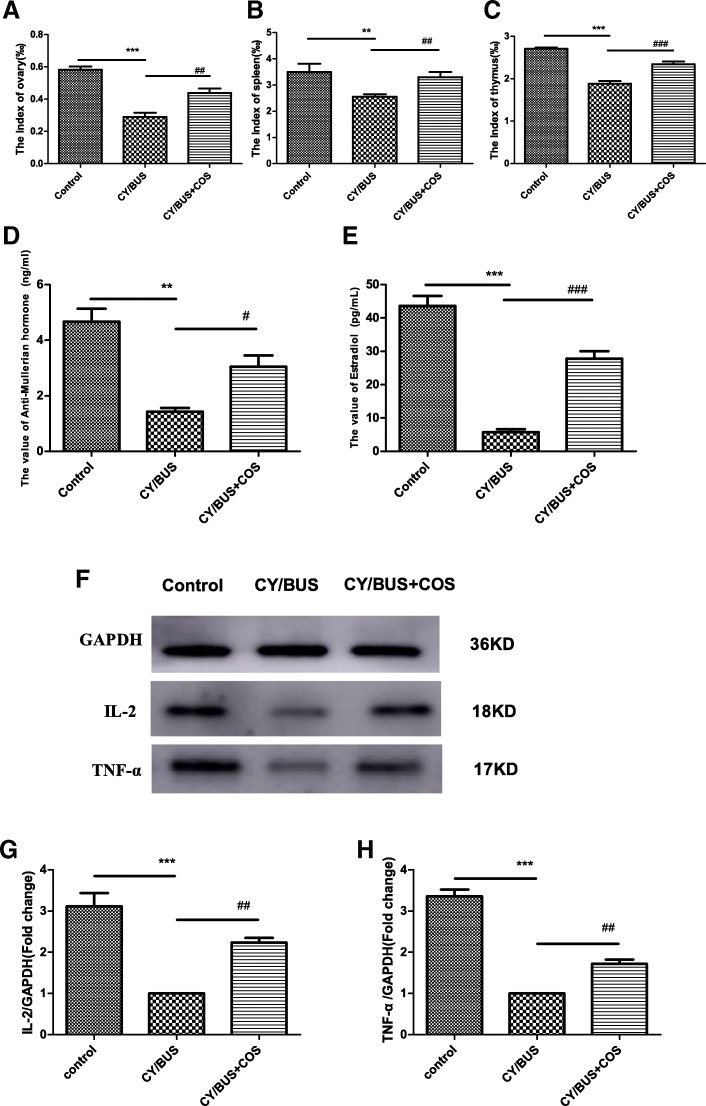
Protective effects of chitooligosaccharides on reproductive and immune function. **a**-**c** The index of different organs in control, CY/BUS and CY/BUS+COS group after 28 days of continuous administration; **d**, **e** Effects of COS on AMH and E2 hormone levels in mice; **f**-**h** Expression of IL-2 and TNF-α protein in ovaries. ** *P* < 0.01, and ***P < 0.001, vs. the control group, ^#^*P* < 0.05, ^##^*P* < 0.01 and ^###^*P* < 0.001 vs. the CY/BUS group

Organ indexes of the ovary, thymus and spleen of mice in the cyclophosphamide/Busulfex model group were significantly lower than those in the control group, while the COS treatment group was able to improve the decreased levels of the ovary, thymus and spleen indexes in mice compared with the CY/BUS group (Fig. 3a-c, Table 4). The results showed that COS increased the weight of ovaries and immune organs in the cyclophosphamide/Busulfex model mice. The levels of AMH and E_2_ in the peripheral blood of mice in the CY/BUS group were significantly lower than those of the control group, while the levels of AMH and E_2_ in the COS group were significantly higher (Fig. 3d, e). The expression levels of IL-2 and TNF-α in the ovaries of the CY/BUS model group were significantly lower than those of the control group, while the COS (300 mg/kg/d) treatment group effectively prevented the decreased levels of IL-2 and TNF-α (Fig. 3f-h).

### Effect of COS on ovarian folliculogenesis in the CY/BUS-induced gonadotoxicity model

**Fig. 4:**
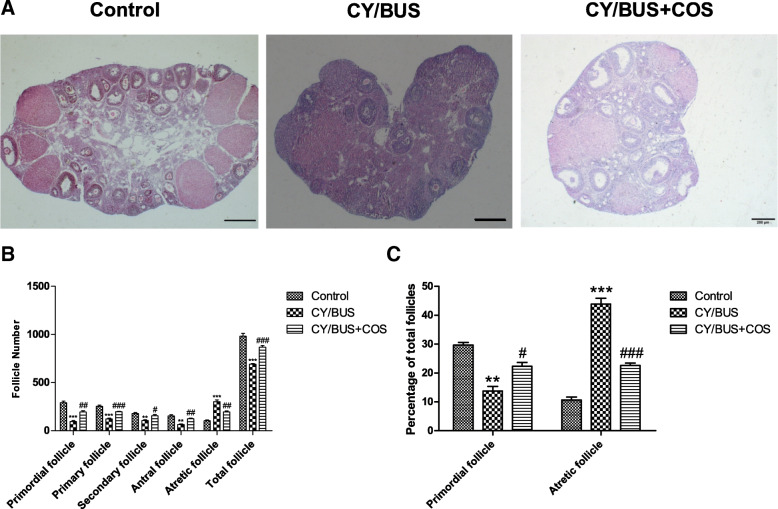
Chitooligosaccharides (COS) administration prevents the loss of different follicle types and decreases atresia caused by cyclophosphamide (CY)/ busulfan (BUS) treatment. **a** Representative images of HE stained sections from control, CY/BUS and CY/BUS+COS mice, the scale bar is 200 μm. **b** The number of follicles at different levels was counted by tissue section. **c** Quantity changes of the percentage of primordial and atretic follicles in total follicles. Data are presented as mean ± SEM for three independent experiments. ** *P* < 0.01, and ***P < 0.001, vs. the control group, ^#^*P* < 0.05, ^##^*P* < 0.01 and ^###^*P* < 0.001 vs. the CY/BUS group

As evidenced by histological assessment, the follicle number of mice in the CY/BUS-treated group was significantly less than that in mice with normal ovaries, as they present obvious cortical fibrosis, while the COS-treated group was similar to healthy, control ovaries (Fig. 4). Counting the number of follicles in all stages showed that the number of primordial (P<0.001), primary (P<0.001), secondary (P<0.01) and antral follicles (P<0.01) were decreased in CY/BUS-treated mice compared to control animals, while atretic follicles were increased (P<0.001), which further showed the chemotoxic effect of CY/BUS and was an ideal modeling method for premature ovarian failure (Fig. 4a). COS treatment elevated the number of primordial and primary follicles compared to the CY/BUS-treated group (P<0.01 or P<0.001, Fig. 4b). The primordial follicle ratio increased (P<0.05) and the number or ratio of atretic follicles dropped (P<0.01 or P<0.001) after COS treatment, compared with the growth in the CY/BUS-treated group (Fig. 4c).

### Identification of ovarian reproductive stem cells

**Fig. 5:**
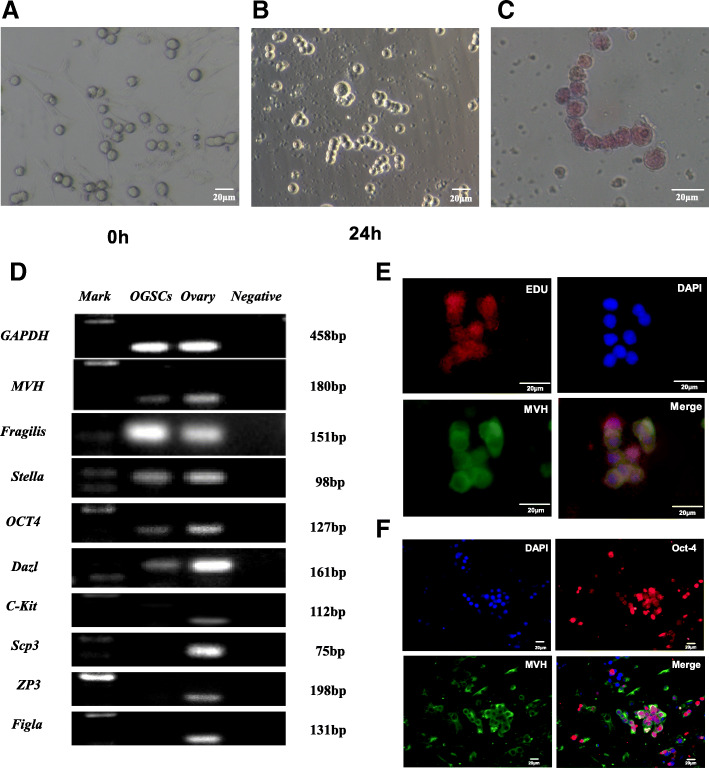
Identification of ovarian reproductive stem cells. **a**, **b** Morphology of ovarian germline stem cells in early stage of culture; **c** Alkaline phosphatase staining; **d** Identification of ovarian germline stem cells and whole ovary by RT-PCR; (E,F):Immunofluorescence of Ovarian Germline Stem Cells

To verify whether OGSCs have the characteristics of germ cells and stem cells, a series of identification experiments were carried out on the OGSCs in culture. The germline stem cells isolated from the ovary were round and had large nuclei of approximately 10–20 μm in diameter. The number of freshly isolated stem cells was small; however, but after 24 h of self-proliferation and division, the number of cells increased continuously and finally showed a beadlike shape, which was similar to the growth pattern of spermatogenic stem cells (Fig. 5a, b). Alkaline phosphatase (ALP) test results showed that OGSCs were weakly positive (Fig. 5c). mRNA of ovarian germ stem cells and the whole ovary after 7 days was extracted to detect the expression of related markers by RT-PCR, and the results showed that OGSCs expressed MVH (germ cell specific marker), fragilis (germ cell specific marker), DAZL (germ cell specific marker), OCT4 (stem cell specific marker) and Stella (markers of pluripotent cells and germ cells) (Fig. 5d). MVH, OCT4, and EdU (cell proliferation marker) were used for simultaneous fluorescence double-labeling. The results showed that OGSCs were all expressed, confirming that OGSCs were proliferative ovarian reproductive stem cells, rather than formations of multiple cells adhering to one another (Fig. 5e, f).

### The distribution of chitooligosaccharides in cells at different times

**Fig. 6:**
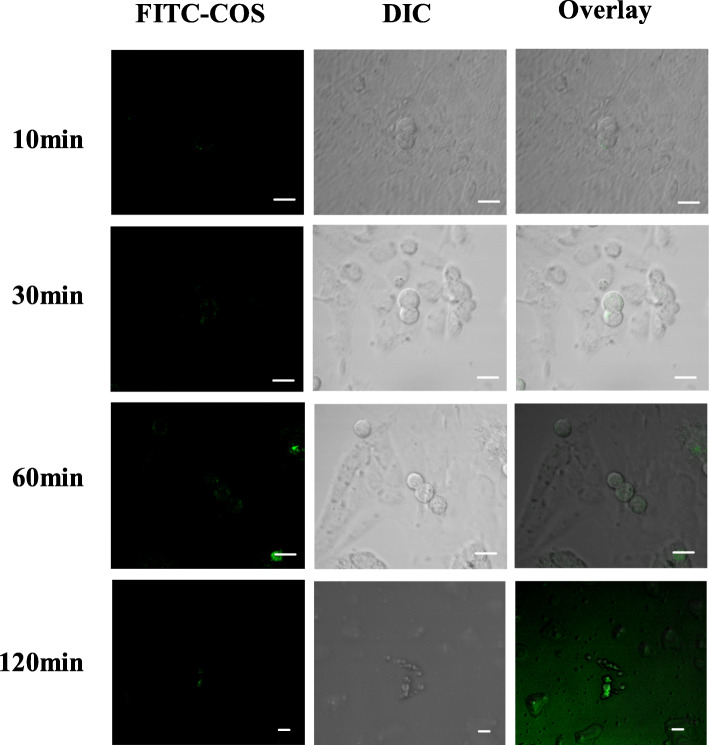
The distribution of FITC-COS into OGSCs changed with time (Bar:10 μm)

FITC-COS (50 μg/mL) and OGSCs were cultured in a CO_2_ incubator at 37 °C for 10 min, 30 min, 60 min and 120 min, respectively. With the extension of the culture time, the fluorescence intensity of the cells changed. When cultured for 10 min, some cells showed a small amount of fluorescence in a granular distribution. Thereafter, as the incubation time increased, the fluorescence intensity of the cells gradually increased, showing the time dependence (Fig. 6).

### Distribution of FITC and COS in OGSCs

**Fig. 7:**
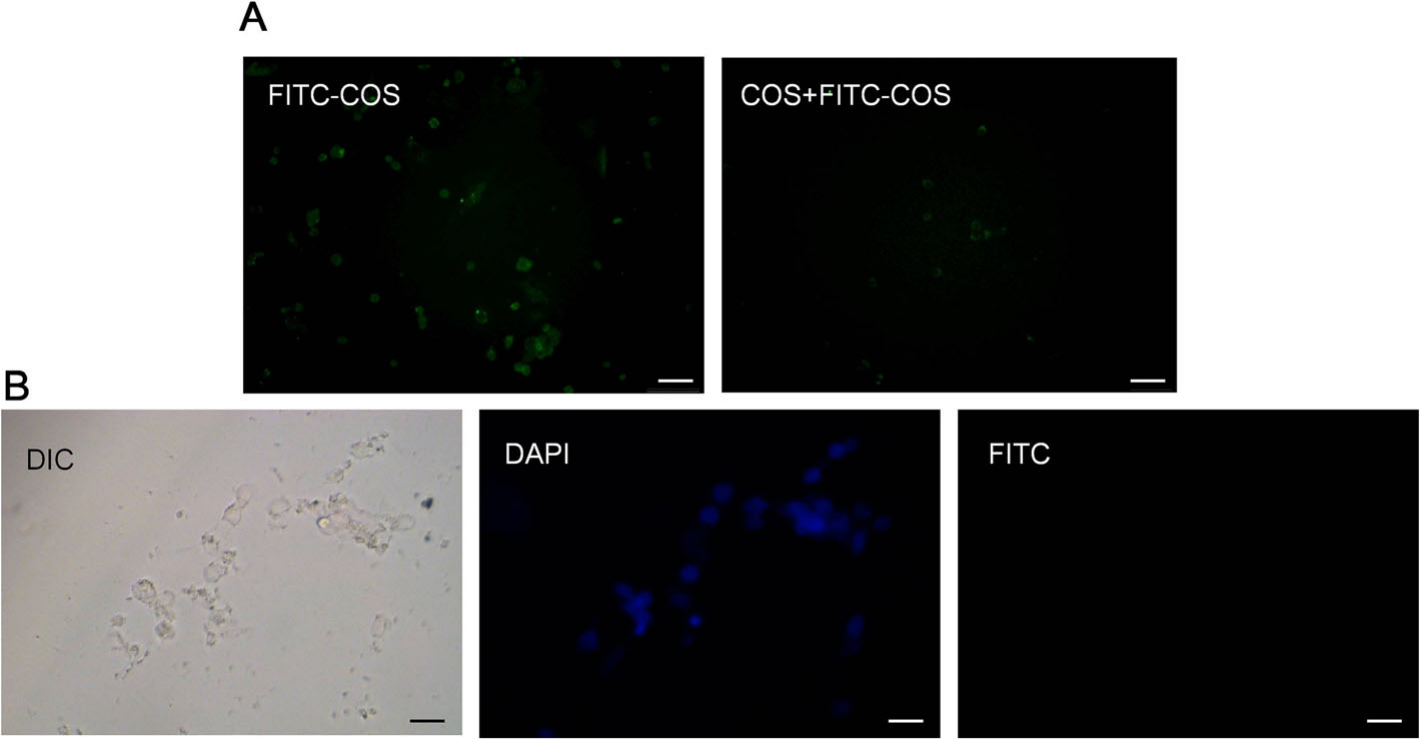
Distribution of FITC and COS in OGSCs. **a** After 1 h, the fluorescence distribution of FITC-COS group and COS + FITC-COS group in OGSCs was observed. **b** Fluorescence distribution of FITC in OGSCs (Bar:20 μm)

In order to explore whether COS entered OGSCs, we first cultured OGSCs with COS for 30 min, and then added FITC-COS for 30 min. The results showed that compared with FITC-COS direct culture group, the fluorescence intensity of OGSCs decreased significantly, indicating that COS entered the cells and had a saturation effect (Fig. 7a). At the same time, FITC was cultured with OGSCs, and the results showed that FITC did not adhere to the cells in a non-specific way (Fig. 7b).

### Effects of cytokines IL-2 and TNF-α on OGSC proliferation

**Fig. 8:**
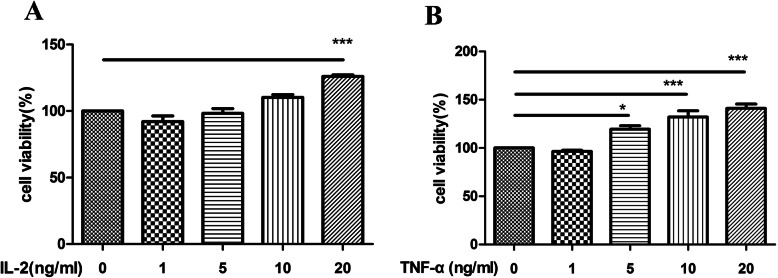
The proliferation effect of different concentrations of cytokines on OGSCs. **a** The cytokine IL-2; **b** The cytokine TNF-α. *p < 0.05, ***p < 0.001, vs. the control group

Cytokines IL-2 and TNF-α at various concentrations (1 ng/ml, 5 ng/ml, 10 ng/ml, and 20 ng/ml) were cultured with OGSCs for 72 h (combined with previous laboratory results), and the proliferative activity of each group was measured using CCK-8. Compared with the control group,the IL-2 group (20 ng/ml) and TNF-α group (10 ng/ml and 20 ng/ml) had a significant effect on OGSC proliferation (Fig. 8a, b). The results indicated that the cytokines IL-2 and TNF-α significantly promoted OGSC proliferation.

## Discussion

Poor living environment, enormous work pressure and other factors are important reasons for the occurrence and development of female infertility. Effective prevention and treatment of ovarian dysfunction caused by chemotherapy is the focus of this paper. The immune regulatory network plays an important role in the development of many diseases, and inflammatory microparticles are closely related to the diseases in organs, such as the heart, brain, kidney and skin [36–38]. The decline in ovarian function is no exception. For women, ovarian function is greatly affected by external factors. The expression levels of inflammation-related genes in the ovaries of mice with decreased ovarian function are significantly higher than those of the ovaries of mice in the fertile period. This change in the microenvironment may lead to a decline in gamete quality with the environment changes [39]. Studies have shown that the levels of TNF-α and IL-2 in the serum of patients with decreased ovarian function are significantly lower than those of the normal group, and the expression levels of IL-1β, IL-6, IL-10 and TGF-β are upregulated in ovaries with decreased function [40]. Germline stem cells (GSCs) are adult stem cells responsible for gamete production, while OGSCs have the function of proliferation and division. Previous studies showed that there are multiple signaling pathways involved in the proliferation and differentiation of OGSCs, and the occurrence of the decline of ovarian function maybe associated with the loss of OGSCs. Supplementation of OGSCs may have a role in remodeling ovarian function [41–43]. These findings have important implications for the reproductive capacity of female mammals and the treatment of germ cell-related diseases, and provide new directions for the treatment of ovarian function decline after chemotherapy for scientists. COS is a natural immune enhancer with no toxic side effects, good biodegradability, adhesion and compatibility, and has been widely used in drug delivery, immune stimulation, tissue regeneration and wound healing [44–46].

To analyze the differences in tissue distribution of COS through different administration routes to the ovary in vivo, FITC and COS were chemically linked in this experiment, FITC-COS with a labeling rate of 3.5% was prepared, and the prepared samples were confirmed to have no free FITC fluorescein by HPLC. The study shows that the labeling rate of approximately 4% has no effect on the structure and properties of COS, which can better reflect the distribution of substances in the body [28]. In this experiment, fluorescently labeled FITC-COS was administered to mice through the abdominal cavity, gavage and tail vein. Ovarian tissues were taken at specified times to make tissue homogenates, and absorbance was measured and substituted into the FITC-COS standard curve. The concentration of FITC-COS can be calculated by FITC-COS standard curve. The results showed that the retention time to the ovary by intraperitoneal injection was the longest, and the fluorescence value could be detected from 30 min to 12 h. Although the arrival time of tail vein injection was the earliest, the fluorescence value gradually decreased with increasing time, while the retention time and drug concentration to the ovary by intragastric administration of COS were not as good as those of the previous two methods. It may be due to the fact that tail vein injection does not require absorption and first pass effect, and the drug utilization rate is 100%. Intraperitoneal injection has two absorption pathways, one of which is the portal vein as the main absorption pathway. Drugs first pass through the liver and then distribute to the whole body. Therefore, a small number of drugs have the first pass effect through the liver, reducing the amount of drugs entering the systemic circulation. Oral administration is mainly absorbed through the gastrointestinal mucosa and blood is absorbed through the small esophageal intestine and small intestinal capillaries. After inactivation and metabolism of some drugs through the intestinal mucosa and liver, the amount of drugs entering the systemic circulation decreases. By contrast, the intraperitoneal administration method has a large peritoneal area, dense blood vessels and lymphatic vessels, strong absorption capacity, extremely high local drug concentration, and very long maintenance time, which can directly contact the abdominal viscera without considering the cardiac load, compared with intravenous injection operation, which is convenient. Therefore, the retention rate will be different in different administration methods. Combined with the experimental results, the intraperitoneal administration mode was chosen to continue the subsequent experiments.

The ovary is the reproductive organ of female animals. Whether the structure of the ovary is complete, the number of follicles in the ovary and whether the primordial follicles can develop and mature all determine the reproductive life of the female [47]. The pathogenesis of decreased ovarian function after chemotherapy remains unclear. At present, most scholars think that the decline of immune system function may change the microenvironment of OGSCs nest, affecting the proliferation and differentiation of OGSCs, and then leading to the depletion of the primordial follicular pool [48, 49]. To explore the rescue effect of COS intraperitoneal injection on CY/BUS-induced ovarian function decline in mice, a CY/BUS-induced gonadotoxicity mouse model was established. After 21 days of continuous administration, corresponding ovarian and immune indexes were observed. From the HE results, it can be seen that the ovarian structure of the CY/BUS group of mice was almost completely changed, with a large number of follicles reduced, and the ovaries presented interstitial fibrosis. However, normal follicles could be seen in the ovaries of mice in the COS treatment group, and the recovery effect was good. AMH plays an important role in gonadal development, and is produced by ovarian granulosa cells in women; its hormone levels gradually decrease with age. AMH is a good index for evaluating ovarian reserve function, which can suggest a low ovarian response; it is not affected by the menstrual cycle and can be detected at any time [50–52]. E_2_ is produced by the ovary and placenta, which is responsible for regulating the stability of the female body environment and stimulating the development of follicles. Its concentration affects the release of gonadotropins, thereby affecting ovarian function [53, 54]. By examining the serum sex hormone levels, we found that the levels of AMH and E_2_ were extremely low in the CY/BUS group, and the hormone levels were significantly greater after COS treatment, further confirming that COS reversed the pathological ovarian hypofunction caused by CY/BUS modeling. Some studies found that the ovaries of nude mice with congenital thymus defects had poor development and shortened reproductive life spans. When antithymocyte serum was administered intraperitoneally to normal adult female mice, the corpus luteum of the ovaries of mice persisted and stopped ovulation [17, 55]. Consistent with this phenomenon, the ovary, thymus and spleen of the CY/BUS model were atrophied to varying degrees in our experiment, while the ovary, thymus and spleen index recovered to some extent after COS treatment. To further explore the role of the immune system in ovarian function decline, we detected the expression of the immune factors IL-2 and TNF-α in ovaries by WB, and the results showed that COS might reversed the decrease in the expression of two immune factors caused by CY/BUS. According to the literature and experimental results, COS improved the ovarian microenvironment by regulating the immune system, thus remodeling ovarian function.

Since Johnson et al. first discovered the production of new oocytes from female mammals in 2004, they questioned the “primordial follicular pool fixation theory.” Tilly et al. isolated OGSCs from the ovaries of normal women and implanted them into transgenic adult female mice, and observed the formation of new primordial follicles. More and more researchers have found the existence of OGSCs in mammalian ovaries and studied it deeply [4, 56, 57]. Our laboratory also successfully isolated and cultured OGSCs from the ovaries of suckling mice. The cells expressed several specific markers of germ cells and stem cells such as MVH, fraglis, DAZL, OCT4 and Stella by RT-PCR, and were positive by MVH/OCT4 and MVH/EDU fluorescence double-labeling and alkaline phosphatase staining, which again suggested that the isolated OGSCs had the characteristics of germ cells and stem cells.

The ovarian surface is covered with a single layer of cubic or flat epithelial cells, Wu et al. [6] isolated OGSCs expressing germ cell characteristics from suckling mice and adult female mice using a magnetic bead screening method, and successfully localized them in the epithelial layer of the ovary. In this experiment, the distribution of COS in the ovary was observed by intraperitoneal administration of FITC-COS to mice. The distribution of COS in the kidney tissue was used as a control. It was found that COS was mainly distributed in the epithelial layer of the ovary, which was consistent with the distribution of OGSCs in ovary. While in the kidney, COS was mainly distributed in the parenchyma. Early studies referred to this as ovarian germ stem cell nest, because the previous results of the OGSC laboratory showed that COS had a certain direct promoting effect on the proliferation of OGSCs, but the effect was not significant. To explore whether the improvement of ovarian function by COS was also related to the direct and intracellular substance action of OGSCs, our laboratory observed the combination between COS and OGSCs by laser confocal microscopy. The experimental results showed that the combination of COS and OGSC is very fast. A small amount of fluorescence can be observed on the cell membrane in 10 min. With increasing time, the fluorescence intensity of the cells gradually increases, showing the time dependence. Since COS is water-soluble, it is considered that COS may enter the cell by facilitated diffusion or by phagocytosis. However, the current results cannot confirm that COS has entered the cell, and it may only be absorbed on the cell membrane. Therefore, we need to further use technologies such as laser confocal microscope and flow cytometer to study the sub-localization of COS in cells and the specific mechanism of action.

Immune-related cytokines are important components in the microenvironment of OGSC nests, which participate in and affect the asymmetric division of OGSCs, promote the formation of dominant follicles, and thus maintain the normal physiological function of ovaries [17, 55]. Previous laboratory studies have found that using macrophages as feeder layer, COS can significantly promote the proliferation of OGSCs and inhibit their senescence. The supernatants of cells were assayed, and the results showed that the secretion of the immune cytokines IL-2 and TNF-α increased. TNF-α is produced by activated monocytes/macrophages, that promote cell proliferation and differentiation and also synergize with epi-epidermal growth factor and insulin to promote the expression of the EGF receptor and produce growth factor-like effects on some tumor cells [58, 59]. Studies have shown that [60] there is a biphasic activity of TNF-α in the follicles before ovarian ovulation, which not only inhibits the production of prostaglandin F2a (PGF2a), but also stimulates the temporary elevation of PGF2a before ovulation to promote the occurrence of ovulation. IL-2 is an interleukin produced by T cells or T cell lines. It stimulates T cells to secrete B cell proliferation and differentiation factors, promoting thymocytes to enter S phase after activation by antigen stimulation, and then maintaining cell proliferation. Some studies have shown that [61] IL-2 and IL-18 combined stimulation can effectively promote NK cell proliferation, IL-18 induces NK cells to enhance IL-2R, especially CD25, to make cells respond to IL-2, activating STAT3 and STAT5, increasing cyclin B1 expression and thus promoting cell proliferation. Therefore, it is speculated that COS might promotes the proliferation of OGSCs by increasing the secretion of TNF-α and IL-2.

To further explore whether the proliferation of OGSCs is regulated by immune cytokines, CCK-8 experiments were designed. The results of CCK-8 assays showed that TNF-α and IL-2 at various concentrations (1 ng/ml, 5 ng/ml, 10 ng/ml, 20 ng/ml) interfered with the cells, and the results showed that both cytokines promoted the proliferation of OGSCs. These results are consistent with those of previous laboratory studies. In the preliminary results of the laboratory, we preliminarily explored the effect of COS on the proliferation of OGSCs. The experiment was divided into four groups: control group (group 1),COS group (group 2), and RAW 264.7 group (Group 3) and RAW 264.7 + COS group (Group 4) to explore whether there is indirect effect. The data of 1–4 groups showed that the cell proliferation curve showed different degrees of increase, in which the cell supernatant cytokine detection found that IL-2, TNF - α showed different degrees of increase. Immune factor (IL-2, TNF - α) related proteins and ovarian germ stem cell (MVH, Oct4) related proteins were increased in varying degrees. Combined with the preliminary laboratory results, it is suggested that COS may have a certain proliferative effect on OGSCs; however, but it mainly relies on promoting the secretion of immune cytokines to promote the proliferation of OGSCs indirectly.

In summary, through the study of this subject, COS was successfully combined with FITC using a chemical method and free fluorescein was removed. The optimal administration mode of COS to the ovary was screened using spectrophotometry and HPLC technology. The remodeling function of COS in pathological ovaries was confirmed by follicle counting, serum sex hormone level, organ index and other methods. In addition, the observation of the combination process of COS and OGSCs using laser confocal microscopy provides a preliminary basis for future studies on the sublocalization of COS within cells. Further studies in cell experiments confirmed that COS indirectly promoted the proliferation of OGSCs by regulating the secretion of the immune factors IL-2 and TNF-α, thereby improving ovarian function. These results might provide a new idea and method for further study of specific immune regulation mechanisms, sublocalization of COS combined with OGSCs and its mechanism of action, and whether COS can maintain local concentration stability and increase through targeted nanotechnology, laying a theoretical and experimental foundation.

## Conclusion

In conclusion, this study enriched the potential mechanism of delaying ovarian function decline. We found that COS could promote the proliferation of OGSC and reshape the ovarian function by improving the ovarian microenvironment and stimulating the secretion of immune related factors. In the future, we will construct the ovarian targeted COS nano delivery system, and observe the effect of targeted nano COS on the proliferation and differentiation of OGSC and the recovery of damaged ovaries through in vitro organ and cell level. The molecular mechanism of COS delaying ovarian function decline is still unclear, which needs further basic research to prove.

## Data Availability

Some or all data used during the study are available from the corresponding author by request.

## Abbreviations

COS: Chitosan oligosaccharide
mRNA: Messenger ribonucleic acid
IL-2: Interleukin-2
TNF-α: Tumor necrosis factor-α
FITC: Fluorescein isothiocyanate
OGSCs: Ovarian germline stem cells
RB200: Ethylrhodamine B
TRITC: Tetramethyl rhodamine isothiocynate
BSA: Bovine serum album
FBS: Fetal bovine serum
DEPC: Diethyl pyrocarbonate
PBS: Phosphate buffered saline
PCR: Polymerase Chain Reaction
RT-PCR: Chain Reaction
HE: Hematoxylin eosin
POI: Premature ovarian insufficiency
TGF-β: Transforming growth factor-β
Stra8: Stimulated by retinoic acid gene 8
AMH: Anti-mullerian hormone
E2: Estrogenic hormone
HPLC: High performance liquid chromatography
EDTA: Ethylenediaminetetraacetic acid
CCK-8: Cell Counting Kit-8
OD: Optical density
